# Impact of Bariatric Surgery on Adipose Tissue Biology

**DOI:** 10.3390/jcm10235516

**Published:** 2021-11-25

**Authors:** Óscar Osorio-Conles, Josep Vidal, Ana de Hollanda

**Affiliations:** 1 Centro de Investigación Biomédica en Red de Diabetes y Enfermedades Metabólicas Asociadas (CIBERDEM), Instituto de Salud Carlos III (ISCIII), 28029 Madrid, Spain; oosorio@clinic.cat; 2 Translational Research in Diabetes, Lipids and Obesity, Institut d’Investigacions Biomèdiques August Pi I Sunyer (IDIBAPS), 08036 Barcelona, Spain; 3 Obesity Unit, Endocrinology and Nutrition Department, Hospital Clínic de Barcelona, 08036 Barcelona, Spain; 4 Centro de Investigación Biomédica en Red Fisiopatologia de la Obesidad y Nutrición (CIBEROBN), Instituto de Salud Carlos III (ISCIII), 28029 Madrid, Spain

**Keywords:** bariatric surgery, adipose tissue, obesity, subcutaneous adipose tissue, visceral adipose tissue, cytokines, adipokines, adipocyte

## Abstract

Bariatric surgery (BS) procedures are actually the most effective intervention to help subjects with severe obesity achieve significant and sustained weight loss. White adipose tissue (WAT) is increasingly recognized as the largest endocrine organ. Unhealthy WAT expansion through adipocyte hypertrophy has pleiotropic effects on adipocyte function and promotes obesity-associated metabolic complications. WAT dysfunction in obesity encompasses an altered adipokine secretome, unresolved inflammation, dysregulated autophagy, inappropriate extracellular matrix remodeling and insufficient angiogenic potential. In the last 10 years, accumulating evidence suggests that BS can improve the WAT function beyond reducing the fat depot sizes. The causal relationships between improved WAT function and the health benefits of BS merits further investigation. This review summarizes the current knowledge on the short-, medium- and long-term outcomes of BS on the WAT composition and function.

## 1. Introduction 

White adipose tissue (WAT) has evolved to become the largest endocrine organ. Its plasticity in response to excess or deficit of nutrients is crucial to maintain metabolic health. The remodeling and expansion capacity of adipose tissue implies the orchestrated response of adipocytes, immune cells, endothelial cells, fibroblasts, the extracellular matrix, and its secretome (cytokines, hormones, microRNAs) as mediators of crosstalk between the main organs involved in metabolic health. Dysfunctional expansion of adipose tissue emerges as a key determinant of obesity-related complications. WAT expansion beyond the subcutaneous adipose tissue (SAT) capacity leads to visceral adipose tissue (VAT) expansion and ectopic fat deposition in other tissues, which are major contributors to cardiovascular disease and metabolic risk above body mass index (BMI) [1]. The precise mechanism leading to impaired adipose tissue expandability are not fully understood. Bariatric surgery (BS) currently results in weight loss and better control of comorbid obesity conditions than medical therapy. BS is also associated with a reduced risk of mortality and of some types of cancer [2]. Currently, Roux-en-Y gastric bypass (RYGBP), sleeve gastrectomy (SG), and biliopancreatic diversion (BPD) are the main surgical techniques used worldwide [3].

This review aims to delve into the biology of adipose tissue in the context of obesity and its changes after BS.

## 2. Obesity-Related White Adipose Tissue Dysfunction

To identify what hypothetical benefits BS has on adipose tissue biology, we need to cite first the most consensed features of obesity-related WAT dysfunction: an altered adipokine secretome [4,5], unresolved inflammation [6,7], inappropriate extracellular matrix (ECM) remodeling, and insufficient angiogenic potential [8]. The causal order in this context is not completely known; however, hypertrophic adipocytes seem more prone to this scenario as they reach the diffusional limit of oxygen, resulting in persistent hypoxia and ultimately leading to unhealthy WAT tissue expansion [8]. Given its role in WAT remodeling, some authors add autophagy dysregulation to this context [9,10]. Among these features, inflammation-related phenomena, i.e., impaired adipokine and cytokine secretion, have been undoubtedly the most exhaustively studied and tracked parameters during the postsurgical follow-up period after BS.

Obese WAT is characterized by macrophage infiltration, a condition considered as both the cause and consequence of its immune response, which leads to chronic inflammation [11,12]. Obesity-related accumulation of adipose tissue macrophages (ATMs) has been clearly demonstrated in multiple studies [13,14,15] and the majority of such ATMs accumulate in omental rather than subcutaneous depots [14,15,16]. Thus, while a small number of macrophages, preferentially localized near blood vessels and dispersed among mature adipocytes are found in lean WAT, subjects with severe obesity show a higher abundance of infiltrating macrophages forming crown-like structures (CLS) around single adipocytes [13]. Such macrophages predominantly present the M1 pro-inflammatory phenotype and promote inflammation by releasing tumor necrosis factor alpha (TNF-α) and interleukin 6 (IL-6), thus contributing to insulin resistance. Alternatively activated, M2-like macrophages play a role in WAT expansion, thermoregulation, antigen presentation, and iron homeostasis [17]. In lean humans, the number of M2 ATMs predominates, secreting anti-inflammatory cytokines and utilizing oxidative metabolism to maintain WAT homeostasis. During obesity development, their proportion compared to M1 ATMs decreases and both populations may adopt a glycolytic metabolism [18]. Once the WAT healthy growth capacity is exceeded, the production of specific adipokines and cytokines by adipocytes and ATMs is compromised and can affect other organ systems.

Although the secretome of many other pro- [19,20,21] and anti-inflammatory [19,22,23] mediators have been found altered in the context of obesity, those described here are the most comprehensively evaluated, and their postsurgical modulation at different follow-up times is summarized below. 

### 2.1. Adipose Tissue-Derived Cytokines

Although the systemic impact of WAT cytokine production in the context of obesity and diabetes has been recently called into question [24], it has been consistently shown that BS-induced weight loss progressively decreases the infiltration of macrophages and WAT secretion of pro-inflammatory molecules [25]. Such cytokines can be both released by WAT-resident immune cells or directly from adipocytes. Below, we describe the most well-studied in the context of obesity.

#### 2.1.1. Pro-Inflammatory

TNF-α is a 17 kDa pro-inflammatory cytokine that can be secreted from mature adipocytes but is predominantly produced within WAT stromovascular fraction, including preadipocytes, endothelial cells, smooth muscle cells, fibroblasts, leukocytes, and macrophages [26,27,28]. The latter is thought to be the major responsible for the elevated expression during obesity [29].

IL-1β is a major 17.5 kDa pro-inflammatory cytokine secreted mostly by macrophages [30], and its release from WAT nonfat cells is augmented during obesity [31]. With other inflammatory mediators, its production is greater in the visceral than in the subcutaneous depot [32]. Increased circulating IL-1β levels have been associated with the risk of developing type 2 diabetes [33], inasmuch as IL-1β contributes to inhibiting β-cell function and destroying β-cell mass [34,35] and impairs adipocyte insulin signaling [36].

Adipocytes, fibroblasts, and endothelial and immune cells secrete IL-6, which induces fever and liver production of the acute phase reactants, and also mediates chronic inflammatory responses [37]. Both adipocytes and macrophages are responsible for its overexpression in WAT during obesity [38,39]. Visceral rather than subcutaneous depot seems to be the main source of circulating IL-6 levels [40]. 

Different cell types express IL-8, such as monocytes, macrophages, fibroblasts, endothelial cells, and adipocytes [41,42]. IL-8 acts as a chemokine, attracting leukocytes [39]; as a pro-angiogenic factor [43]; and an amplifier of inflammation [44]. Secretion of IL-8 from WAT is increased [45], mainly in the visceral depot [37] during obesity, and is associated with insulin resistance [42].

IL-18 is another pro-inflammatory cytokine, produced by both hematopoietic cells and non-hematopoietic cells, which has been found to be increased in obesity [46,47] and associated with the metabolic syndrome independently of obesity and insulin resistance [48].

Chemoattractant chemokine ligand 2 (CCL2), also referred to as MCP-1, is a chemoattractant cytokine produced by, among others, myeloid cells [49] and adipocytes [50]. The latter enhances MCP-1 secretion during obesity [51], recruiting and activating macrophages through the MCP-1/IL-1β/CXCL12 signaling pathway [52]. Nevertheless, WAT expansion augmentation does not influence circulating MCP-1 levels [51].

Three isoforms of transforming growth factor-beta (TGF-β) have been identified in mammals, which are produced by all-white blood cells lineages and, to a lesser extent, by mature adipocytes [53]. Despite originally being thought to have overlapping functions, isoform-specific knockout mouse models revealed non-redundant phenotypes [54,55,56], TGF-1β being the predominant and most important isoform [57]. TGF-1β release from WAT is enhanced in obesity and in response to insulin and inhibitors of TNF-α and IL-β and correlates BMI and adiposity [53].

#### 2.1.2. Anti-Inflammatory

Secreted by Th2 T-cells, M2 macrophages, and adipocytes [39,58], IL-10 is an anti-inflammatory cytokine that suppress macrophage activation [59], which has been inversely associated with BMI and body fat content [60].

Primarily secreted from mast cells and eosinophils, cytokines IL-4 and IL-13 are closely related, where the former stimulates the production of the latter [61], both sharing similar anti-inflammatory functions and receptor complexes [62]. The presence of these cytokines promotes alternative activation of macrophages into M2 cells and inhibits M1-like classical activation [63]. Both IL-4 [64] and IL-13 [65] serum concentrations are increased in obesity. Moreover, recent research showed a role for IL-4 in promoting adipocyte thermogenic capacity [66] and lipolysis [67] through hormone-sensitive lipase (HSL) modulation [68].

### 2.2. Adipose Tissue-Derived Hormones 

#### 2.2.1. Pro-Inflammatory

Mostly produced by adipocytes, leptin is a highly conserved 167 kDa peptide. It is secreted proportionally to the amount of adiposity [69,70]. Leptin acts to reduce the food intake at the level of the hypothalamus and fat stores at the level of the adipocyte [71], as well as promoting pro-inflammatory cytokine production by immune cells [38,58].

Resistin, traditionally considered a WAT-specific secretory factor, is a 12.5 kDa hormone which acts as a modulator of body cholesterol trafficking, increasing low-density lipoprotein (LDL)-cholesterol and degrading liver LDL receptors, thus contributing to atherosclerosis pathogenesis. Within WAT, resistin promotes pro-inflammatory cytokine production through the resistin receptor and is found to be increased in obesity [72]. Nevertheless, mounting evidence reveals inconsistencies between resistin’s role in rodents and humans, and its relationship with insulin resistance in humans is still controversial [73], with arguments existing both for [74,75,76] and against [77,78,79] this association.

Visfatin is another proinflammatory adipokine that plays a role in insulin sensitivity and whose production is increased in obesity and correlates with visceral adiposity [80].

#### 2.2.2. Anti-Inflammatory

Adiponectin is secreted from WAT as an oligomer of varying sizes in an inversely proportional manner to the degree of visceral adiposity [81]. Adiponectin plays an anti-inflammatory role and promotes insulin sensitivity by increasing fatty acid oxidation, thus regulating lipoprotein metabolism and inhibiting hepatic glucose production. The adiponectin-leptin ratio is considered a biomarker of inflammation in WAT [82,83]. 

Predominantly expressed in the visceral depot [84], omentin -34 kDa- is an anti-inflammatory adipokine with insulin-sensitizing effects whose levels are decreased in obesity and diabetes [85] and inversely correlated BMI [86]. The role of other molecules such as apelin, vaspin, and RBP4 in inflammation is less clear.

### 2.3. Extracellular Matrix Remodeling and Fibrosis

WAT is a highly dynamic organ, as it is responsible for storing and releasing energy in response to nutrient excess or shortage. As WAT expands (by adipocyte enlargement—hypertrophy; and preadipocyte recruitment—hyperplasia), ECM is remodeled to accommodate healthy WAT expansion. Like in other organs, sustained WAT inflammation can trigger aberrant ECM deposition leading to WAT fibrosis. Profibrotic mediators such as TGF-β or connective tissue growth factor (CTGF) participate in this pathway [87]. When WAT becomes fibrotic, ECM stiffness impedes healthy remodeling, causing the tissue to be metabolically dysfunctional, displaying, e.g., adipocyte death, decreased lipolysis, and disrupted cell–cell interactions [87,88]. Thus, inflammation can disarrange the tight balance between ECM composition, extracellular metalloproteinases (MMPs), and their inhibitors (TIMPs) [89]. Data from three independent studies carried out by Karine Clément’s group in BS subjects identified the degree of fibrosis in SAT as a predictor for poorer weight loss response after BS [90,91,92]. In this context, HIF1α has been proposed to link the hypoxic milieu to fibrosis and inflammation [93]. Certainly, accumulating evidence demands further research on the relationship between multiple ECM components and adipocyte function in the context of obesity [88,94,95,96,97] and diabetes [98,99,100], and possible associations with BS outcomes should be explored in depth.

### 2.4. Basal and Stimulated Lipolysis

Obesity is associated with an increase in basal lipolysis and impaired insulin ability to suppress the FFA outflow [101,102]. Antagonistically, plasma catecholamines are important stimulators of lipolysis via adrenergic receptors, particularly through beta-1 (ADRB1) and beta-3 adrenergic receptors (ADRB3) in human WAT [103], and catecholamine-stimulated lipolysis has also been found to be impaired in obesity [104]. Although the classical notion of ‘catecholamine resistance’ in obesity seems to receive little attention today, some authors recommend its revisitation [105]. 

More than two decades ago, Kaartinen et al. found a good correlation between the fat cell size and response to isoproterenol in isolated SAT adipocytes from subjects with obesity undergoing BS [106]. Interestingly, after substantial BS-induced weight loss, the lipolytic effect of isoproterenol stimulation of adrenergic receptors was higher than lean controls, despite no difference in receptor density between groups. Similar results have been reported after short-term nutrition interventions [107,108]. Fasting FFA circulating levels are other relevant measures of basal lipolysis, though they are not only dependent on WAT lipolysis but also on clearance by muscle and the liver. 

### 2.5. Angiogenesis

Adipogenesis and angiogenesis are tightly related processes during ‘healthy’ WAT expansion since adipocyte differentiation trigger blood vessel formation [109,110], and in turn, WAT endothelial cells promote preadipocyte differentiation [111]. Vascular endothelial growth factor (VEGF)-A, highly expressed in WAT, plays a capital role in angiogenesis, and its expression is raised during adipogenesis [112,113]. Besides the family of VEGF factors, the angiopoietin (ANGPT) family is also involved in vascular remodeling, maturation, and stabilization [114]. ANGPT-2, expressed in WAT endothelial cells, is considered a proangiogenic factor. Although its overexpression in mice improved the metabolic status [115], its role in the angiogenic process has not yet been elucidated. Platelet endothelial cell adhesion molecule-1 (PECAM-1/CD31) plays a role as an adhesion and signaling molecule with several roles in vascular and inflammatory processes, and its levels are increased in young men with severe obesity [116].

### 2.6. Autophagy

Autophagy, the cellular mechanism that promotes cell survival during nutrient depletion, may also be relevant under basal or nutrient excess conditions. During nutrient depletion, autophagy can provide essential components for energy production and biosynthesis. In circumstances of nutrient excess, autophagy plays an important role in eliminating unfolded proteins and toxic aggregates and facilitating endoplasmic reticulum homeostasis [117]. In this regard, liver autophagy has been the subject of extensive research [118], while WAT autophagy has been receiving growing attention in recent years and is now considered a key regulator of adipogenesis [9] with intricate implications in ECM remodeling and inflammation [10]. Although some authors have reported attenuated WAT autophagy in obesity [119], not all studies could confirm the sense in which obesity and/or metabolic disruption is related to WAT autophagy alterations [10], and most studies point to overactivation of WAT autophagy in obesity [120,121,122] and diabetes [123,124]. However, several considerations should be taken into account [10]. Since WAT autophagy can be regarded as a protective mechanism to avoid WAT maladaptation to nutritional stress, this may explain enhanced autophagy despite the increased inflammation in dysfunctional WAT. In addition, autophagy has different functions depending upon the cell type; thus, WAT cell heterogeneity should be taken into consideration. Finally, the varied technical approaches used to measure autophagy and the different depots analyzed could explain conflicting results among these studies. All of this together calls for much more research into the relationship between autophagy, obesity, and BS outcomes.

## 3. Bariatric Surgery—Related Changes in White Adipose Tissue Biology

Since there is no standardization and the definition of short-, mid- and long-term terminologies can vary among published reports [125], from here on, the current knowledge on this topic is summarized across five follow-up time points commonly used to report BS outcomes: ≤3 months (3 m), 6 m, 1 year (1 y)—all often considered to be short-term; ≥2 y <5 y—referred to as medium-term; and >5 y—frequently regarded as long-term post-surgery. All bariatric interventions considered in Table 1 consisted of SG, RYGB, or BPD.

### 3.1. Short Term

During the first year, coinciding with the rapid weight loss phase after BS, both SAT [126,127,128,129,130] and VAT [126,127,128,129,130,134,135] depots progressively reduce their size, and this is accompanied by a reduction in the area of subcutaneous [136,137,138] and visceral [138] adipocytes, respectively. A large adipocyte size was independently associated with a lower incidence of insulin resistance 6 months after RYGBP [136].

In the very short term after BS (≤3 months), Cancello et al. showed a significant decrease of total ATMs (HAM56+ cells) in SAT after RYGB [13]. These results were confirmed in another study from the same group, wherein CD40^+^ cells (M1-like) were also found to be decreased and CD206^+^ and CD163^+^ cells (M2-like) increased 3 months after RYGB [16]. This was accompanied by a reversion to the lean WAT profile, with CLS remission and ATMs again located near blood vessels [13].

Such early changes in the WAT cellular composition seem to alter the production of some cytokines, while others generate conflicting results between studies or do not seem to be modulated in the short term after BS. Thus, among the proinflammatory cytokines, MCP-1 was found to be concomitantly decreased during this period [13,150,165,166], while TGF-β or IL-1β seem to decrease only at 1 year after BS [142,147,156]. Reports on IL-6 production give conflicting results at 3 and 6 months but agree on a consistent decrease 1 year after surgery [139,142,143,144,146,150,152,156,158,159,160,168]. Similarly, reduced circulating levels of IL-18 were found 1 y post-BS [141] and after massive BS-induced weight loss, irrespective of the time elapsed since surgery [195,196].

In contrast, there is less consensus about TNF-α and IL-8, which have been found in different studies to both be increased [140,155,156,197], decreased [141,142,143,144,163,164], or unchanged [146,147,148,149,150,151,152,157] during this period. Similarly, BS-related outcomes on anti-inflammatory cytokine production have yielded highly contradictory results between studies during the short-term follow-up period, as is the case with IL-4 [64,140,167], IL-10 [141,147,162,164,167,168], and IL-13 [144,167]. Interestingly, circulating omentin levels decrease as early as 24 h post-BS, before any fat mass loss, and maintained for 1 y [172].

Inasmuch as surgical weight loss predominantly reduces the body fat content, it is understandable that leptin levels were found to be consistently reduced following BS [140,141,144,148,149,150,156,158,162,163,168,170]. The leptin levels were also reduced after the novel endovascular bariatric procedure [198]. Nevertheless, systemic leptin levels are not directly related to the amount of body weight or fat loss, since early reductions of adiposity more dramatically reduce leptin levels than later periods of weight loss [162,170]. Again, there is a lack of consensus regarding the short-term effect of BS on resistin levels, given several studies have found it to be decreased [141,142,145,158,168] or unchanged [150,171]. In the case of visfatin, Lima et al. showed unaltered levels throughout the first year after BS [150].

Despite some conflicting reports in the very short term [146,147,149,150,156], circulating adiponectin levels appear to be consistently increased 1 year after BS [139,141,142,150,152,156,158,168]. For its part, omentin was found to be increased as early as 24 h after BPD [172], and such a change is maintained for up to 1 year [172,173]. Apelin, a multifaceted biomarker [174], and vaspin, an insulin-sensitizing adipokine [175], are less investigated adipokines that showed a short-term reduction after BS. Regarding RBP-4, most studies reported a decrease in the circulating [177,178] or SAT mRNA [176] levels early after BS.

One study performed by Chabot and collaborators showed no resolution of SAT fibrosis 6 months after BS and suggested a transient association between SAT fibrosis and insulin resistance in humans with obesity [180]. Similarly, Katsogiannos et al. did not find significant differences in either the basal or stimulated lipolysis rate in SAT adipocytes at 1 and 6 months after BS but reported a decrease in isoproterenol-stimulated lipolysis at 6 versus 1 month after BS [181]. Conversely, insulin-suppressed free fatty acid (FFA) release has been found to be enhanced at 4 months [137], 7 months [183], and 1 year after RYGBP [101]. While some authors found increased FFA levels in the early months after BS [101,148,181], others reported no differences in this period [148,184,185,186]. 

García de la Torre et al. found higher VEGF-A levels in obese women undergoing BS compared to lean controls, and such levels significantly decreased 1 y after surgery, irrespective of the surgical procedure performed [187]. At this same follow-up period, another recent study showed, in addition to VEGF-A, lower levels of several angiogenesis biomarkers such as angiopoietin 2 (ANGPT-2), follistatin, hepatocyte growth factor (HGF), and the platelet endothelial cell adhesion molecule (PECAM-1) in patients who underwent SG or laparoscopic adjustable gastric banding (LAGB) [188].

Finally, Soussi et al. found attenuated WAT autophagy in obesity, and pre- versus post-BS comparisons indicated ameliorated adipocyte autophagic clearance in all patients within 3 to 12 months after the intervention, although at different degrees because of the large time-frame in post-surgery sample collection [119]. 

### 3.2. Medium Term

Two years after surgery, both visceral and subcutaneous depots maintain reduced sizes [126,131,132] as does the abdominal subcutaneous fat cell volume [132]. There is much less data available on circulating parameters beyond 1 y after BS. While IL-6 levels are consistently found reduced 2 y [145,153], 3 y [154], and 4 y after BS [169], reports on TNF-a continue to report conflicting data [145,154]. Although reports on IL-10 also seem quite inconsistent, some authors find that, after a temporary rise in the short term, its levels return to baseline values at 2 y [145], or even continue falling at 4 y [169]. 

BS outcomes on leptin and adiponectin levels seem much more solid. Circulating leptin has been repeatedly found to be reduced at 2 [145,153], 3 [154], and 4 y [169], and such reductions seem to be mainly attributed to early changes in WAT. Conversely, adiponectin levels continue to progressively rise in the medium term [145,153,154]. Only one report seems to oppose this view, a contradiction that could arise from the limited number of subjects and the variety of surgical techniques included in the study [169]. 

Beyond the short-term inconsistencies mentioned above, a single study showed that circulating resistin, after an early decline, recovered baseline levels 2 y after gastric bypass [145]. Finally, the RBP-4 levels were found still lowered 24 months after BS. Such changes were more pronounced in the subgroup without metabolic syndrome and correlated with reductions in the waist and visceral fat diameter [179].

Despite negative results reported by Katsogiannos et al. in the short term in a mixed-sex cohort [181], Löfgren and collaborators found reduced basal and stimulated lipolysis rates at 2 y after BS exclusively in females [182], where differences in the basal rates remained only significant when lipolysis was expressed per cell surface area. In another study, the glycerol release in women who underwent RYGBP was found to be decreased postsurgically at 2 y and then increased dramatically to similar levels observed before surgery at 5 y [133]. Similarly, Manco et al. found reduced FFA levels in normoglucose-tolerant obese women 3 years after BPD [154]. Finally, insulin-mediated suppression of FFA outflow has been found to be enhanced 3 years after RYGBP [102].

### 3.3. Long Term

Studies on long-term outcomes after BS are restricted almost exclusively to weight-loss parameters. Thus, a recent meta-analysis at 10 or more years after all bariatric procedures reported weighted means of 56.7% excess weight loss (EWL) after GB, 45.9%EWL after LAGB, 74.1%EWL after BPD and 58.3%EWL after SG [199]. The same study reported a 48.9%EWL and 22.2%TWL 20 y after LAGB. Very similar results were previously reported by the same group at 15 y after LAGB [200]. A lower incidence [201] and greater remission [202] of T2DM have also been reported in the long term; reductions in all-cause, cardiovascular, and T2DM mortality have also been found [203]. Nevertheless, the potential impact of body fat loss on these metabolic outcomes deserves further investigation since some variables appear to be more weight-dependent, while others seem to be more adiposity-dependent from the medium-term [204].

Regarding the outcomes in WAT exclusively, we only have evidence from a single study carried out in women by Hoffstedt and collaborators at the long-term follow-up [133]. The authors reported decreased amounts of estimated SAT and VAT at 2 and 5 y and diminished SAT cell volume and increased adiponectin levels at 5 y post-BS. This study also found augmented basal glycerol release from isolated SAT adipocytes at 5 y, despite not finding changes in fasting plasma levels.

### 3.4. Summary of BS Outcomes on WAT

In summary, after bariatric surgery, SAT and VAT reduce their size progressively during the weight-loss phases. M1-like decrease and M2-like ATMs increase early after surgery; however, there are no data beyond the short term after BS. 

Most pro-inflammatory cytokines begin to decrease early after surgery and continue to decline in the medium- and long-term. However, TGF-B or IL1B decrease only after one year of BS. There are controversial data on short-term TNFα and IL-8 levels after surgery as well as in anti-inflammatory cytokine levels in the short- and medium-term after surgery. Leptin levels drop rapidly soon after BS and then continue to decline during the follow-up; conversely, adiponectin and omentin levels rise after surgery. Resistin and visfatin dynamics show less agreement.

Regarding fibrosis, only one study reported no changes at short-term. Gender differences seem to affect basal and stimulated rates of lipolysis, which have been found decreased only in females at mid-term after BS. For its part, insulin inhibition of lipolysis was found consistently enhanced at medium- and long-term after surgery. Finally, autophagy increases and several angiogenesis-related molecules decrease at short-term, although there is a lack of reports on longer follow-up periods.

## 4. Other Proposed Novel Mechanisms for WAT Improvement after BS

Mitochondrial function and biogenesis have been found to be impaired in obesity, and T2DM and BS may attenuate mitochondrial damage in adipocytes. Thus, Varela-Rodríguez and colleagues reported an increased mitochondrial density and coverage, together with enhanced mitochondrial function at both the gene and protein level in abdominal SAT after RYGB- or SG-induced weight loss in a reduced cohort of patients [205]. In the very short term after RYGB, an induction of genes involved in mitochondrial biogenesis was found in SAT [206]. Similarly, increased SAT expression of transcripts related to the oxidative phosphorylation (OXPHOS) pathway has been shown 3 m [207] and 1 y after BS [137,208]. More recently, Van der Kolk et al. confirmed these findings in abdominal SAT in the short and medium term after RYGB, while opposite results were found after a low-calorie diet [209], suggesting a BS-specific effect. The authors also showed an induction of the tricarboxylic acid (TCA) cycle and fatty acid oxidation 2 y after surgery.

Beiging is the process through which WAT can change its phenotype to a brown-like adipose tissue known as beige/brite adipose tissue. Accumulating evidence from human and rodent studies in the last years suggest that RYGB predominantly enhances beige thermogenesis, while SG seems to promote brown adipose tissue thermogenesis [210]. A role for bile acids and the gut microbiome has been proposed in these mechanisms. Moreover, such thermogenic effects could depend on the fat content of the postoperative diet.

Beyond the fat mass loss and biological pathways discussed above, other mechanisms could contribute to the improvement of WAT dysfunction after BS. Thus, Frikke-Schmidt and colleagues recently summarized other potential pathways affecting the WAT function that can play a role after BS [105]. Bile acids, whose levels are persistently found to be increased after surgery [211], may improve adipocyte function acting upon the FXR receptor [212]. Another hypothesized mechanism implies gut microbiome composition. It has been found that the composition of the bacteria in the gut change after BS, and accumulating evidence shows how the bacterial composition can modulate the host immune cell population. Thus, there is the possibility that changes in the gut microbiome initiated by BS can significantly impact the WAT metabolic function by modulating immune-resident cells in WAT [213,214]. Finally, as BS has demonstrated effects on the central regulation of metabolism, potential changes in neural enervation to the WAT may mediate physiological changes after BS, as results in mice suggest [215].

Lastly, recent studies have indicated significant alterations in the expression of several putative adipose tissue-derived microRNAs (miRNAs) after BS [216]. Thus, 1 y after BS, Sangiao-Alvarellos et al. reported raised circulating levels of miR-221 and miR-222 [190], and as early as 21 days post-RYGB, Atkin et al. found modulated levels of seven miRNAs (miR-7-5p, let-7f-5p, miR-15b-5p, let-7i-5p, miR-320c, miR-205-5p, and miR-335-5p) [189], mostly related with diabetes and insulin resistance pathways. Regarding SAT expression, Ortega and collaborators identified 12 modulated miRNAs 2 y after BS, some of them previously found raised in mature adipocytes after inflammatory stimulation (such as miR-146b, miR-376c, and again miR-221) [194], while another study from the same group found significant modifications in 15 mature miRNAs, mostly related to cell cycle, metabolism, and inflammation pathways, in women who underwent RYGB [191].

## 5. Future Perspectives

Better understanding of the fascinating biology of WAT following BS deserves further investigation. Evaluation of the modifications of WAT biology associated not only with time elapsed after surgery but also with the amount of weight loss is a priority. Studies should help us better understand the relationship between shrinkage in WAT volume and improved WAT function with the health benefits of BS. The health burden associated with a particular BMI in subjects with a weight-reduced state following BS appears to be eased as compared to that in subjects with comparable BMI that have not undergone BS. Thus, future studies should help disentangle how BS helps restore the crosstalk between the different components of the WAT as well as the crosstalk between WAT and other organs.

## Figures and Tables

**Table 1:** Short-, medium- and long-term outcomes of bariatric surgery on fat depot parameters, circulating and adipose tissue expression levels of cytokines, adipokines, and microRNAs.

	Short-Term						Medium-Term		Long-Term	
Parameter	≤3 m		≈6 m		1 y		≥2 y		≥5 y	
Depot size										
Subcutaneous	↓	[126]	↓	[126,127,128,129]	↓	[126,127,130]	↓	[126,131,132]	↓	[133]
Visceral	↓	[126,134]	↓	[126,127,128,129]	↓	[126,127,130,135]	↓	[126,131,132]	↓	[133]
Fat cell area										
Subcutaneous	-		↓	[136,137]	↓	[137,138]	↓	[132]	↓	[133]
Visceral	-		-		↓	[138]	-		-	
Proinflammatory cytokines										
TNF-α	↑	[139,140]	↓	[141]	↓	[142,143,144]	↑	[145]	-	
	=	[146,147]	=	[148,149,150]	=	[146,148,150,151,152]	=	2 y [153], 3 y [154]	-	
					↑	SAT [155]				
IL-1β	=	[147]	-		↓	[142]	-		-	
IL-6	=	[147,150,156]	=	[141,149,150,157]	↓	[139,142,143,144,150,152,156,158,159,160]	↓	[24]	-	
	↓	[140,156,161]	↓	[139,160,162], SAT [148]	-		-		-	
IL-8	=	[147]	=	[157]	=	[152]	-		-	
	↑	[156]			↓	[163]				
	↓	[161,164]								
IL-18	-		-		↓	[141]	-		-	
MCP-1	↓	SAT [13]	↓	[164,165]	↓	[150,164,166]	-		-	
TGF-β	=	[156]	-		↓	[156]	-		-	
Anti-inflammatory cytokines										
IL-4	=	[140]	↑	[167]	-		-			
			↓	MNC [64]						
IL-10	=	[147]	↑	[141,167]	↑	[168]	=	2 y [145], 4 y [169]	-	
			=	[162]	↓	[164]	-			
			↓	[164]						
IL-13	-		↑	[167]	↓	[144]	-		-	
Proinflammatory adipokines										
Leptin	↓	[140,149,162,170]	↓	[141,148,149,150,162,170]	↓	[144,148,150,156,158,163,168]	↓	2 y [145,153], 3 y [154], 4 y [169]	-	
Resistin	=	[150]	↓	[141]	↓	[142,158,168]	↓	[145]	-	
			↑	[150]	=	[150,171]				
Visfatin	=	[150]	=	[150]	=	[150]	-		-	
Anti-inflammatory adipokines										
Adiponectin	↑	[147,149,156]	↑	[139,141]	↑	[139,141,142,150,156,158,168]	↑	2 y [145,153], 3 y [154]	↑	[133]
	=	[146,150,156]	=	[150,162]			=	4 y [169]		
Omentin	↑	[172]	↑	[172]	↑	[172,173]	-		-	
Other adipokines										
Apelin	-		↓	[174]	-		-		-	
Vaspin	-		-		↓	[175]	-		-	
RBP-4	↓	SAT [176]	↓	[177,178]	↑	[151]	↓	[179]	-	
	=	[176]								
Fibrosis										
Subcutaneous	-		=	[180]	-		-		-	
Lipolysis										
Basal	=	[181]	=	Isolated SAT adipocytes [181]	-		=	Male [182]	=	[133]
							↓	Female [133,182]	↑	SAT release [133]
Stimulated	=	Isolated SAT adipocytes [181]	↓	Isolated SAT adipocytes vs. 1 m [181]			=	Male [182]	-	
							↓	Female [182]		
Insulin-supressed	-		↑	[137,183]			↑	[102]	-	
FFA	↑	[148,181]	=	[148,184,185]	↑	[101]	=	[102]	-	
	=	[186]			=	[148]				
Angiogenesis										
VEGF-A	-		-		↓	[187,188]	-		-	
ANGPT-2, follistatin, HGF, PECAM-1	-		-		↓	[188]	-		-	
Autophagy										
Subcutaneous	-		↑	3–12 m post-BS [119]	-		-		-	
microRNAs										
	↑ ↓	7 Circulating miRNAs [189]		-	=	Circulating miR-99b [190]	↑ ↓	15 SAT miRNAs [191]	-	
	↓	1 VAT and 13 SAT miRNAs [192] *			↑	Circulating miR-221, miR-222 [190]	↓	SAT miR-221-3p [193]		
							↓	12 SAT miRNAs [194]		

For cytokines, adipokines, lipolysis, and angiogenesis markers, data refer to circulating levels, unless otherwise stated. ↑, Increased; ↓, decreased; =, equal or inconclusive; -, no data; m, months; y, years; TNF-α, tumor necrosis factor-α; IL, interleukin; MCP-1, monocyte chemoattractant protein-1; TGF-β, transforming growth factor β; RBP-4, retinol binding protein 4; FFA, free fatty acids; VEGF-A, vascular endothelial growth factor A; ANGPT-2, angiopoietin-2; HGF, hepatocyte growth factor; PECAM-1, platelet endothelial cell adhesion molecule-1; SAT, subcutaneous adipose tissue; VAT, visceral adipose tissue; MNC, mononuclear cells. * after significant weight loss, collection time not reported.
